# Supplementary material for: Factors associated with post-neonatal mortality in Ethiopia: Using the 2019 Ethiopia mini demographic and health survey
Source: PLoS One. 2022 Jul 27;17(7):e0272016. doi: 10.1371/journal.pone.0272016 (PMC9328495; doi:10.1371/journal.pone.0272016)
Supplement: Attachment — Submitted filename: Response to reviewer.docx [file pone.0272016.s002.docx]

**Authors' responses to the comments of the Editors and Reviewers**

To begin, I am grateful for your thoughtful review and evaluation of this study, Factors Associated with Post-Neonatal Mortality in Ethiopia: Using the 2019 Ethiopia Mini Demographic and Health Survey. We revised our manuscript in response to the reviewers' comments and suggestions. The following is a point-by-point response to the remarks and suggestions of reviewers and editors.

We trust that we have satisfactorily addressed them and that the manuscript is now ready for publishing**.**

**Response to academic editor suggestions and comments**

**Comment1:** Please ensure that your manuscript meets PLOS ONE's style requirements, including those for file naming.

**Response1:** We appreciate your suggestion. The file names were changed to conform to the style criteria. We should now have no deviations from the style criteria.

**Comment2:** Please amend your current ethics statement to address the following concerns: Did participants provide their written or verbal informed consent to participate in this study?

**Response2:** Thank you for this suggestion. The study used secondary data analysis of publicly available survey data from the DHS program, ethical approval, and participant consent were not necessary for this study. We requested the DHS program, and permission was granted to download and use the data for this study from <http://www.dhsprogram.com.Therefore>, no need for written or verbal informed consent to participate in this study.

**Comment3:** In your Data Availability statement, you have not specified where the minimal data set underlying the results described in your manuscript can be found.

**Response3:** Thank you for this suggestion. *The data in this study were secondary and are available in the public domain at* (<https://www.dhsprogram.com/data>)*. If you need specific data(sample) used in this study, we will submit.*

uploaded minimal data set as a Supporting Information file.

**Comment4:** There are several grammatical errors and typos in the text.

**Response4:** Thank you for your important comments. We repeatedly read the whole document and consider all the corrections and the language is edited by professionals.

**Response to Reviewers’ comments and suggestions**

Dear reviewers, we are contented and appreciate your careful and thorough reading of this manuscript and we would like to say thank you for your thoughtful and constructive comments and suggestions. Based on your comments and suggestions, we carefully corrected the manuscript and addressed every comment one by one. We made rephrasing in some parts of the paragraphs of the manuscript accordingly.

**Reviewer #1**

**Comment**1: The article has lots of punctuation issues (like in the abstract section, line 11,12), which should be corrected in the whole manuscript.

**Response1:** Thank you dear reviewer for your important comments. We repeatedly read the whole document and consider all the corrections.

**Comment**2: As the keywords of the study, the authors should provide more keywords. Also, Binary Logistic Regression is not a good keyword in this regard.

**Response2:** Thank you, dear reviewer, for improving our manuscript. In the revised manuscript, we have made changes based on your feedback.

**Comment**3: The whole instruction part is one paragraph, which might be hard for the reader to follow the clues of the study. The introduction should be divided into two to three paragraphs.

**Response3:** Thank you for your suggestions. Based on your recommendation we divided it into three paragraphs.

**Comment**4: The authors should add two sections at the end of the discussion section. First, they should provide comprehensive data from countries with successful strategies that could effectively control infant mortality. So, these strategies could be used in developing countries. Second, the authors should provide a paragraph for policymakers. This should contain some strategies and concepts for policymakers to be implemented at the society level.

**Response4:** Thank you for your constructive comments. We have incorporated the comments in the revised manuscript at the end of discussion and conclusion parts.

**Comment**5: Providing DOI for references is highly encouraged.

**Response5:** Thank you for your important suggestion. We incorporated DOIs of each reference.

**Reviewer #2:**

**Comment**1: General language editing and revisions regarding grammar and syntax are highly recommended by a native speaker or an expert since there are several grammatical errors and typos in the text.

**Response1:** Thank you for your important comments. We repeatedly read the whole document and consider all the corrections and the language is edited by professionals.

**Comment**2: The methods section in the abstract is vague and needs more details and descriptions

**Response2:** Thank you dear reviewer for your constructive comments. We accepted your comments and gadetailedail explanation in the revised manuscript.

**Comment**3: As mentioned in the Methods section of the study, 2126 “post neonates” were included in the final analyses. However, in the methods of the abstract section, it has been mentioned as 2126 “women aged 15 to 49.” Please make them identical and provide more explicit methods.

**Response3:** We appreciate all of your insightful and critical feedback. It is a critical comment. We went over all of the errors in the manuscript. ETKR (child dataset) with 5753 infants was used. Dear Reviewer! If we were to use the ETIR dataset (women dataset), your suggestion would be the best method to proceed. However, we did not use a women's data set for this study.

**Comment**4: In figure 1, it has been captured that 5753 women were eligible. Please describe in the methods how these women were selected and what the inclusion and exclusion criteria were?

**Response4:** Thank you for your comments. First of all, the data was not women, those 5753 data were child datasets. So, we have made changes. We included inclusion and exclusion criteria in the revised manuscript.

**Comment**5: Please provide more explanations around the data collection process and inclusion and exclusion criteria.

**Response5:** Thank you for your recommendation. We made a change to the revised manuscript and included the points that you have raised.

**Comment**6: Please provide research ethics related to this study in the methods section. Did consent forms obtained from the subjects?

**Response6:** Thank you for your comments. Based on your request, we have included Ethical considerations in the methods section. Since, the study used secondary data analysis of publicly available survey data from the DHS program, ethical approval, and participant consent were not necessary for this study.

**Comment**7: Please explain death in “how many” months after birth was defined as post-neonatal death in the outcome variable.

**Response7:** Thank you for your constructive comments. We have put the specified period on the revised document.

**Comment**8: In the statistical analysis section, please specify and name the tests that were utilized. Were they logistic regression analyses? Please mention them clearly in the methods section

**Response8:** Thank you for these interesting comments. We described it in detail in the revised manuscript.

**Comment**9: When comparing children born with single births to children born with multiple births, the odds of post-neonatal death were.150 (AOR =.150, 95 percent CI: .096, .234) times lower.” What do you mean by “single births and multiple births”? Please define them and make them clear.

**Response9:** Thank you for your comments. We have made changes in the description of independent variables as well as in the result parts.

**Comment** 10: About Minor issues:

**Response 10:** Thank you dear reviewer for all your interesting comments and suggestions. We have read the whole document repeatedly and made revisions for all your minor issues.

**Reviewer #3:**

**Comment**1: The paper needs some editing in English fluency and the draft should become more professional

**Response 1:** Thank you dear reviewer for your constructive comments and suggestions. We repeatedly read the whole document and consider all the corrections and the language is edited by professionals.

**Comment**2: Table 4 has results copied from all presented by SPSS software, I suggest just using practical results in this table with the format that is popular with other published papers.

**Response 2:** Thank you for your interesting comments. We have changed the format of all tables based on references from different published articles.

**Comment**3: About minor issues:

**Response 3:** Thank you for your insightful comments. The issues you identified are critical and have a big impact on the article's quality. We accepted everything and made changes to the updated document.

Sincerely,

On behalf of all authors,

Kenaw Derebe Fentaw
