# Supplementary material for: Factors associated with post-neonatal mortality in Ethiopia: Using the 2019 Ethiopia mini demographic and health survey
Source: PLoS One. 2022 Jul 27;17(7):e0272016. doi: 10.1371/journal.pone.0272016 (PMC9328495; doi:10.1371/journal.pone.0272016)
Supplement: Attachment — Submitted filename: Response to reviewer.docx [file pone.0272016.s003.docx]

**Response to Reviewers’ comments and suggestions**

Again, we are grateful for your thoughtful review and evaluation of this study, Factors Associated with Post-Neonatal Mortality in Ethiopia: Using the 2019 Ethiopia Mini Demographic and Health Survey. Also, we would like to thank you for your appreciation of our work. Based on the comments and suggestions of reviewer #3, we carefully corrected the manuscript and addressed every comment one by one. We made rephrasing in some parts of the paragraphs of the manuscript accordingly.

**Reviewer #1**

**Comment**: I have read the revised version of the manuscript “Factors associated with Post-Neonatal mortality in Ethiopia: Using 2019 Ethiopia mini demographic and health survey”. Overall, all the comments have been addressed and the quality of the manuscript has been improved.

**Response:** Thank you dear reviewer for your acceptance of our revision.

**Reviewer #2:**

**Comment**: Thank you for providing a revised version of this manuscript. Some descriptions regarding data and analyzes were vague in the original version. The authors have reflected on most of the comments and provided more explicit descriptions in the revised version.

**Response:** Thank you dear reviewer for your acceptance of our revision.

**Reviewer #3:**

First of all, we appreciate all of your insightful and critical feedback. All the ideas you raised are very constructive and additional input for the quality of this article.

**Comment**1: As the authors mentioned about the data availability "all data are fully available without restriction", I could not find the specific data regarding this study at <https://www.dhsprogram.com/data> web address. Please provide and upload the dataset you used to analyze.

**Response 1:** Thank you for your interesting comments. As you may know, the DHS dataset is publically available in the aforementioned link address and we extracted our specific data for this study from that one. Based on your request, we have uploaded as additional material in the system the specific dataset that we have used for this study.

**Comment**2: Thanks to the authors for trying to improve the language and remove grammatical errors, but I think just minimal revisions were done, and it can be improved more.
**Response 2:** Thank you for your comment. The language is edited by native speakers and professionals.

**Comment**3: As I commented about revising table 4, I think changes are not enough, because some statistical data are presented which are not necessarily within the scope of this study. For example, why the "degrees of freedom" is necessary to be presented here. Also, other labels are statistical abbreviations, to present it as a medical scientific article, the labels should be changed in this regard.

**Response 3:** Thank you for your insightful comments. The issues you identified are critical and have a big impact on the article's quality. We accepted everything and made changes to the updated document.

**Comment**4: In the abstract part, CI, AOR, and ANC abbreviations are not defined yet.

**Response 4:** Thank you dear reviewer for your comments. We have described these terms in the methods part of the revised manuscript.

**Comment**5: A recommendation: the sentence "Post-neonatal mortality is the number of deaths of infants aged 28 days through 11 months and is expressed as post-neonatal deaths per 1000 live births per year." is too basic and is not necessary enough to be presented in the abstract.

**Response 5:** Thank you dear reviewer for your important comments. If we get your points, we have presented them in the abstract, background, and methodology parts of the manuscript.

**Comment**6: The data gathering time period is not presented in the methods of the abstract part.

**Response 6:** Thank you for these interesting comments. We have made changes to the revised manuscript.

**Comment**7: About the inclusion and exclusion criteria part: some wording issues should be corrected.

**Response 7:** Thank you for these interesting comments. We have made all the corrections.

Sincerely,

On behalf of all authors,

Kenaw Derebe Fentaw
